# Disinfection of Wastewater by UV-Based Treatment for Reuse in a Circular Economy Perspective. Where Are We at?

**DOI:** 10.3390/ijerph18010077

**Published:** 2020-12-24

**Authors:** Maria Cristina Collivignarelli, Alessandro Abbà, Marco Carnevale Miino, Francesca Maria Caccamo, Vincenzo Torretta, Elena Cristina Rada, Sabrina Sorlini

**Affiliations:** 1 Department of Civil Engineering and Architecture, University of Pavia, via Ferrata 3, 27100 Pavia, Italy; marco.carnevalemiino01@universitadipavia.it (M.C.M.); francescamaria.caccamo01@universitadipavia.it (F.M.C.); 2 Interdepartmental Centre for Water Research, University of Pavia, via Ferrata 3, 27100 Pavia, Italy; 3 Department of Civil, Environmental, Architectural Engineering and Mathematics, University of Brescia, via Branze 43, 25123 Brescia, Italy; alessandro.abba@unibs.it (A.A.); sabrina.sorlini@unibs.it (S.S.); 4 Department of Theoretical and Applied Sciences, University of Insubria, Via G.B. Vico 46, 21100 Varese, Italy; vincenzo.torretta@uninsubria.it (V.T.); elena.rada@uninsubria.it (E.C.R.)

**Keywords:** disinfection, ultraviolet radiation, sustainable technologies, wastewater

## Abstract

Among the critical issues that prevent the reuse of wastewater treatment plants (WWTPs) effluents in a circular economy perspective, the microbiological component plays a key role causing infections and diseases. To date, the use of conventional chemical oxidants (e.g., chlorine) represent the main applied process for wastewater (WW) disinfection following a series of operational advantages. However, toxicity linked to the production of highly dangerous disinfection by-products (DBPs) has been widely demonstrated. Therefore, in recent years, there is an increasing attention to implement sustainable processes, which can simultaneously guarantee the microbiological quality of the WWs treated and the protection of both humans and the environment. This review focuses on treatments based on ultraviolet radiation (UV) alone or in combination with other processes (sonophotolysis, photocatalysis and photoelectrocatalysis with both natural and artificial light) without the dosage of chemical oxidants. The strengths of these technologies and the most significant critical issues are reported. To date, the use of synthetic waters in laboratory tests despite real waters, the capital and operative costs and the limited, or absent, experience of full-scale plant management (especially for UV-based combined processes) represent the main limits to their application on a larger scale. Although further in-depth studies are required to ensure full applicability of UV-based combined processes in WWTPs for reuse of their purified effluents, excellent prospects are presented thanks to an absent environmental impact in terms of DBPs formation and excellent disinfection yields of microorganisms (in most cases higher than 3-log reduction).

## 1. Introduction

Water resources are essential for human activities. Due to: (i) the scarcity of water, (ii) the increase in urbanization, and (iii) the discharge into the river of wastewaters (WW) produced by human settlements and industrial activities, proving a correct and adequate water management system is necessary [1]. Water exploitation index (WEI), as established by the European Environmental Agency (EEA) [2], defines water scarcity because it is an indicator of the pressure or stress on freshwater resources [3]. In 2009 Cyprus, Bulgaria, Belgium, Spain, Italy, and Malta were using up 20% or more of their long-term supplies every year and a WEI above 20% implies that a water resource is under stress [4]. In Europe, more than 40.000 million m^3^ of WW is treated every year, but only 964 million m^3^ of this treated water is actually reused; therefore, Europe could use 6 times the volume of treated water that is currently used [5].

In the Circular Economy Action Plan [6], the European Commission referred to the new Water Reuse Regulation in which the reuse of water in agriculture and industrial processes was encouraged [7].

The reuse of treated WW from WWTPs can have a large variety of applications, i.e., irrigation [8], groundwater recharge [9], domestic use [10], industrial applications [11], production of drinking water [10], among others. WW reuse is particularly important in those zones where the water resource is quantitatively and qualitatively scarce [12]. For these reasons and in order to safeguard environmental ecosystems from pollution, in recent years, important to monitor the quality of the WWTP effluent and to define a protocol to evaluate the WW reuse feasibility became important [13].

Among the most critical aspects that prevent water reuse, microbiological contamination plays a key role because microorganisms can cause more or less serious diseases and even death in humans and animals [14]. Several serious infections, such as pneumonia, dermatitis and otitis are the hazardous result of gram-negative bacteria like *Pseudomonas aeruginosa* [15]. In this contest, disinfection represents the main treatment useful for the inactivation of helminths, protozoa, fungi, pathogenic bacteria, and viruses to protect consumers health and the environment making it possible to reuse WWTPs effluents [11,16,17,18]. The most common indicators to assess fecal contamination are *Escherichia coli*, *Enterococcus faecalis* [10,19] and *Faecal coliforms* [19].

Chlorination is one of the most used disinfection methods in WW treatment due to its low cost, management simplicity and its high efficiency in destroying microbes [20,21]. However, its toxicity is widely known [21,22]. In fact, chlorine can generate disinfection by-products (DBPs) by reacting with organic matter present in WW [23,24]. Amid the several classes of DBPs reported in the literature, trihalomethanes (THM) and haloacetic acids, are among the most studied ones [25,26]. The DBPs generated following the use of some chemical oxidants can also be more toxic and dangerous than the starting disinfectant compounds [24,27] having a potential carcinogenicity and other adverse effects on human and animal health also at low concentration [28,29]. For instance, haloacetic acids are considered cytotoxic and genotoxic [30].

Moreover, recent studies proved the potential impacts of chlorination on the transmission of antibiotic resistance genes (ARGs), divided in intracellular ARGs (iARGs) and extracellular ARG (eARG) [31]. Disinfection of antibiotic-resistant bacteria can occur with subsequent release of DNA into the effluent. In this case, eARGs could be absorbed by some bacteria promoting the spread of antibiotic resistance [32]. However, further studies are needed to better investigate the impact of chlorination on these environmental mechanisms occurring from WW.

Necessarily, over the years, other chemicals have been identified as a possible alternative to chlorine in the disinfection of WW, such as chlorine dioxide, ozone and peracetic acid. As regards ozone, among the main disadvantages are the high costs, including energy costs and the operative and management difficulties related to its instability [33]. Ozonation also leads to the formation of bromate as DBP in waters containing the bromide ion (Br^−^) [29,34]. Bromate appears to be a possible human carcinogen and is not biodegradable [35]. On the other hand, chlorine dioxide has an oxidative strength lower than that of chlorine [35] and its use in disinfection treatments can also lead to the formation of DBPs, including chlorite and chlorate which have a negative impact on human health [36]. The formation of unhealthy DBPs (e.g., such as aldehydes and halogenated phenols), even if at limited concentrations, cannot be excluded also using high concentration of peracetic acid [37].

Following these results, it is advisable to develop and implement processes that allow reducing or eliminating the use and generation of substances dangerous for human health and the environment [38,39].

This review focuses on technologies based on ultraviolet radiation (UV) applied individually or coupled with ultrasound, catalysts, and electric current, with the utilization of natural light of the solar spectrum or artificial indoor illumination. In the case of photolysis, both the traditional continuous emission process with mercury lamps and more recent technologies such as UV Pulsed (PUV) and UV-Light Emitting Diode (UV-LED) are described. The strengths in terms of environmental sustainability and above all the most significant critical issues of these applications are presented. The lack of production of unwanted and hazardous DBPs for humans and the environment, unlike chlorination or other oxidation processes involving the dosage of chemicals, allows the achievement of water quality standards for resource recovery and reduces health concerns.

## 2. Methodology and Structure

In order to develop the following review, scientific peer-reviewed literature have been monitored and studied using a multi-stage methodology. The analysis allowed an overall classification of the main UV-based treatments for the reuse of WW. The purpose of the review is to focus on environmentally sustainable processes that minimized the production of unwanted DBPs, toxic and dangerous for humans and the environment and that had a low environmental impact with a reduced waste of resources. Based on these objectives, a series of treatments based on UV rays have been identified: photolysis (UV alone) (including UV pulsed (PUV) and UV light emitting diodes (UV-LED)) and its combination with other technologies: (i) sonophotolysis (UV + ultrasound), (ii) photocatalysis (UV + photocatalyst) and (iii) photoelectrocatalysis (UV + photocatalyst + electric current). The analysis was based on a double screening and control procedure:

Scopus^®^ database has been used to search mainly relevant literature research papers, reviews, and books. The research was divided into four individual research subsections based on the number of treatments considered. In order to find all relevant publications, the keywords used are based on the purpose of the review and identify the concepts of interest. In addition to the name of the individual treatment (“UV radiation”, “UV pulsed”, “UV-LED” for photolysis, “ultrasound” for sonophotolysis, “photocatalysis” for the corresponding treatment and “electrochemical” for photoelectrocatalysis) in the corresponding search, common keywords were used for all processes: “UV”, “disinfection” and “wastewater”. The analysis has been conducted searching the keywords on fields “Article title, Abstract, Keywords”.

- 1° Screening: A preliminary screening of the literature was performed discarding duplicates and only peer-review papers published in English on international journals have been considered. The other publications have been excluded and have not been mentioned in the present review. The selection process continued excluding the articles and reviews that do not analyze sustainable UV-based processes and those which focus on different applications than WW disinfection treatments.
- 2° Screening: The investigation focused mainly on works published in the last 15 years.

**Figure 1:**
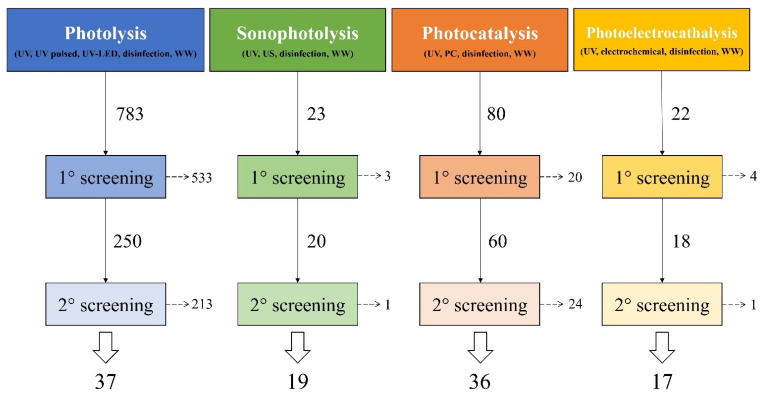
Representation of the multi-step analysis for the screening of literature with the final numbers of reviewed publications. Keywords used in search on Scopus^®^ database are reported in brackets. UV: ultraviolet radiation, WW: wastewater, US: ultrasound, PC: photocatalysis.

The review is based on more than 100 publications that have been divided into the four main selected treatments and categorized depending on the properties and performances described. Figure 1 shows the total number of publications for each treatment and the subsequent screening operations carried out to restrict the evaluation field to only those papers of interest for the point of view of this work.

The review consists of two main sections (Section 3 and Section 4). Section 3 talks about photolysis and the subsections of Section 4 are dedicated to the processes combined with UV. In each paragraph, the relevant characteristics of the corresponding application, including the potentials and above all the limits and critical points, have been reported. In Section 5, discussion on main results and future outlooks of the research are reported. The aim of the review is to deepen the knowledge of these treatments from a critical point of view to have a clearer vision of possible future application options. To focus on some important aspects of the process and solve the critical issues present today, it is advisable to outline the path that researchers will have to follow in their next studies.

## 3. Photolysis

Ultraviolet radiation (UV) is a safe and efficient physical technology for WW disinfection that does not require the use of chemical agents [40] and avoids the production of DBPs [41]. These unwanted intermediaries produced by the reaction of chlorine with the natural organic matter present in WW for example, are linked to the increase in the onset of cancer and other serious diseases for humans [21]. UV, being a physical process, allows to minimize the residue of chemicals dosed for treatment at the WWTP, such as low concentrations of residual chlorine at the discharge which can have toxic effects on aquatic flora and fauna [42]. Therefore, UV disinfection for WW treatment has become an accepted alternative to chemical methods for secondary and tertiary quality effluents, avoiding toxicity problems and ensuring safety for the environment [43,44].

UV includes electromagnetic radiations between the X-rays and visible light in the range of λ from 100 to 400 nm and the germicidal effect of UV-ray is between 220–320 nm [45]. The traditional apparatus consists of continuous-wave mercury lamps in low pressure (monochromatic at 253.7 nm) or medium pressure (polychromatic in the UV and visible light ranges) formats [46]. UV lamps that emit radiation at 254 nm (UV-C) are the ones with the highest germicidal effect [47].

Table 1 presents some studies concerning the application of traditional continuous UV technology on synthetic and real waters. The results show that the highest bacteria reduction values were obtained with synthetic waters consisting mainly of saline solutions with a known initial concentration of bacteria. For example, Zhang et al. [48] obtained the highest inactivation (6-log) in a bacteria suspension in phosphate buffered saline solution with an initial cell density of 10^7^ CFU mL^−1^. However, the reliability of the test is greater if carried out with real water samples from secondary effluents of WWTPs; only by proceeding in this way can results be obtained which better reflect a possible application of the treatment on a large scale.

Solid particles can interfere with the transmission of UV radiation by absorbing or dispersing light and also by adsorbing and/or transporting bacteria, protecting them from the light radiation emitted by the lamps [49]. Therefore, this treatment is more suitable for WW with few suspended solids, otherwise there could also be fouling problems mainly in the quartz lamps sleeves used for the protection of UV lamps [50]. A possible alternative could be the application of further processes (such as the ultrasounds described below) simultaneously with the UV treatment which can reduce the particle size and maintain a high disinfection efficiency [51].

Inactivation of microorganisms by UV light occurs following damage to nucleic acids DNA and RNA: the formation of pyrimidine dimers, other photoproducts and lesions causing the inactivation of the replication and transcription thus preventing the multiplication of cells or viruses [40,52].

Many organisms are known to possess the ability to repair their DNA damage (such as photoreactivation and dark repair), with further bacteria regeneration [53,54]. Pyrimidine dimers in DNA can be repaired in a range of 330–480 nm wavelength (photoreactivation) or they can be repaired without light (dark repair). After the disinfection treatment, the mechanisms of photoreactivation and dark repair can activate the regrowth of the bacteria causing a possible re-proliferation [55]. However, photoreactivation does not appear to be particularly significant at full-scale as it is a mechanism that occurs mainly at low UV doses. Guo et al. [56], found that photoreactivation of *E. coli* (initial concentration: 10^5^ CFU mL^−1^) was not negligible (50%) only for UV dose equal to 5 mJ cm^−2^ and no photoreactivation was detected for UV dose of 15 mJ cm^−2^. In case of total coliforms (initial concentrations: 96,000–250,000 CFU 100 mL^−1^), with a UV dose of 40 mJ cm^−2^, the percentage of photoreactivation was less than 1%. Similar studies were conducted by Hallmich and Gehr [57] and Guo et al. [58].

Considering the energy demand of UV treatment, Fenu et al. [59] reported that the values of the average specific energy consumption on the full-scale were in the range between 0.04–0.13 kWh m^−3^.

In recent years, to reduce some weaknesses of conventional UV and increase disinfection performance, research is focusing on two different approaches: (i) UV Pulsed (PUV)—UV-Light Emitting Diode (UV-LED) or (ii) integrating the UV treatment with non-chemical technologies.

### 3.1. UV Pulsed UV (PUV) and UV-Light Emitting Diode (UV-LED)

#### 3.1.1. UV Pulsed (PUV)

UV Pulsed (PUV) can be an alternative to the traditional application of continuous wave UV irradiation with mercury lamps. It is typically obtained with a xenon lamp (therefore mercury-free) but still presents few research applications to WW disinfection. Fitzhenry et al. [64] analyzed the impact of suspended solids on the inactivation efficiency of *E. coli* through two UV disinfection mode: flow-through PUV and continuous low-pressure UV disinfection. The results indicated that the flow-through PUV system appeared to be more sensitive to the presence of organic suspended solids in WW samples. Therefore, the low-pressure UV seemed to be a more robust disinfection method. Although the study conducted by Bohrerova [60] showed a greater disinfection efficiency of the PUV lamp in a synthetic water, to date, whether PUV systems can be more/less effective in reducing microorganisms in water compared to conventional UV in continuous light is not clear. This happens both because there is no univocal and standard method to compare the results of the two technologies and because more in-depth studies are needed to confirm the effectiveness of PUV lamps in WW. For example, Uslu et al. [65] investigated the effectiveness of continuous-flow PUV light for the inactivation of *E. coli* and *Bacillus subtilis* spores in synthetic effluents and real urban WW. The results showed that in synthetic waters complete inactivation was observed at a flow rate of 10 L min^−1^ for *E. coli* (Chamber volume: 2.9 L, Resident time: 18 s, Total broadband energy: 115.2 J) and a flow rate of 6 L min^−1^ for *B. subtilis* (Chamber volume: 2.9 L, Resident time: 29 s, Total broadband energy: 187.2 J). Testing real waters complete inactivation was observed with a flow rate of 10 L min^−1^ for *E. coli*, while a reduction of 4.15-log was observed at 6 L min^−1^ for *B. subtilis*. Other results on the application of PUV irradiation are reported in Table 2.

#### 3.1.2. UV-Light Emitting Diode (UV-LED)

The most used equipment in water treatment systems is mercury UV lamps which, in addition to the high energy consumption, have a series of critical issues such as fixed wavelengths, low durability, limited cycling and mercury toxicity [71]. One possible solution could be to use ultraviolet light emitting diodes (UV-LED) which are compact, shock resistant, energy-efficient and has a long life (100,000 h) [70]. Therefore UV-LED have been emerging in recent years as a possible source for the generation of UV radiation [67,72,73]. Further studies are needed to investigate the aspect related to the inactivation of bacteria to affirm the better or similar efficiency of UV-LED compared to the other UV technologies described. Zou et al. [74] reported the inactivation of *E. coli* in water applying PUV and continuous UV-LED, using a high power 285 nm LED and low power 265 and 280 nm LED. High current pulsed irradiation of 280 nm LED showed remarkable inactivation enhancement (about 3-log) compared with continuous irradiation (about 2.5-log).

UV-LED can also be a valid alternative as it can be turned on and off with a high and adjustable frequency [72]. In the study conducted by Song et al. [72], the inactivation of *E. coli* bacterium and MS2 virus in synthetic laboratory waters and of *E. coli* and total coliforms in real WW was investigated, by continuous and pulsed irradiation, using UVLED. The results showed comparable inactivation of all microorganisms examined by continuous and pulsed UV-LED irradiation at 265 nm under equivalent UV fluence. So continuous and pulsed irradiation appears to be used to achieve comparable inactivation, but pulsed irradiation can ensure better thermal management for high UV-LED performance. This aspect can represent an advantage over the PUV irradiation of conventional xenon lamps described in the previous paragraph. Table 2 also shows the results of some studies conducted mainly in recent years, demonstrating that UV-LED technology is one of the innovative and emerging ones.

## 4. Combination with Other Technologies

### 4.1. Sonophotolysis

One of the limits of WW disinfection by UV is represented by turbidity because the high concentration of suspended solids can reduce the efficiency of the treatment [51]. Bacteria find protection in solid particles and become resistant to disinfection. One solution is represented by a pre-treatment upstream of the UV process to reduce the particle size which results in the combination of UV and ultrasound (US) technology [51,75]. The combination of US and UV irradiation is commonly known as sonophotolysis. US is a chemical-free way that improves disinfection kinetics, reduces the necessary UV dose demand, inhibits the formation of fouling, and removes fouling from the UV lamps [76,77]. US consists of the transmission of sound waves at frequencies (from 18 kHz to 500 MHz) outside of human hearing ability with successive wavelengths of 10–0.01 cm [78] and low-frequency US (<100 kHz, typically 20–48 kHz) is typically used for the inactivation of microorganisms in WW effluents [76]. The use of US in WW is based on the acoustic cavitation which causes both chemical and physical/mechanical effects on WW compounds [79]. This acoustic phenomenon consists first in the formation of microbubbles and then in growth and violent collapse of cavities in a liquid [75,78,80]. After collapsing, the microbubbles generate H_2_O_2_ and hydroxyl radicals such as H^•^ and OH^•^, which are able to inactivate enzymes and damage membranes, DNA, RNA, proteins and lipids and are responsible of oxidation processes [81]. Besides the chemical effect [76], physical effects, associated with an increase in temperature (pyrolysis and combustion) and pressure, is generated causing the mechanical disruption of the cell membranes [82,83]. The sonication, decreasing the particle size distribution [84], provided more chance for UV irradiation to reach the pathogenic bacteria and improved the disinfection efficiency, but the main drawback of this technology is the high energy consumption [77]. Zhou et al. [85], in their research with a baffled US/UV disinfection reactor at a pilot scale, demonstrated that US pretreatment or simultaneous US/UV disinfection could improve the disinfection efficiency with 0.4 and 0.5 log compared with UV disinfection alone without increasing the specific energy consumption. Even in the experiments conducted by Blume and Neis [86], with a US pretreatment, there was a growth from 0.8 log units to 1.2 log units compared with the samples that were not pretreated.

Lab-scale experiments have shown that larger WW particles (90–250 µm) tend to break more easily than smaller ones (38–63 µm diameter) and as such require less power [75,87]. Zhou et al. [68] reported that the particles larger than 50 µm could be reduced from 63% to 5% using a US power density of 30 W L^−1^ for 30 s. For a 50% reduction in 100 μm WW particles requires from 400 to 1500 J L^−1^ of US energy [75,88].

Naddeo et al. [80] conducted a series of tests with a pilot-scale plant using a plug-flow US and UV simultaneous disinfection reactor continuously operated in a WWTP for four days. Results showed that *E. coli* concentrations in disinfection effluents could respect the limit set for WW reuse in Italy (10 CFU 100 mL^−1^) when a US unit with a dose of 1400 W and a UV unit with a dose of 1656 mJ cm^−2^ was applied for 15 min. However, Zhou et al. [85] stressed that this system requires process optimization due to the high operation costs with a specific energy consumption of about 5.35 kWh/m^3^. To obtain a high log reduction of microorganisms by ultrasound irradiation it is necessary to use high intensities, and this is the limiting economic factor [79].

Furthermore, the energy costs related to the disinfection of water with the US are a few thousand higher than those of the UV process, therefore on a large scale the costs for the additional treatment to the US seem to be economically unjustified [51]. Zhou et al. [77] reported that there is a nonlinear relationship between US energy demand and reactor size, so specific energy consumption or operative cost per unit could be significantly reduced for large-scale practical applications compared to the pilot scale. In their study with a system power input of 270 W (two 85 W UV lamps + one 100 W US transducer), a specific energy consumption of 0.225 kWh m^−3^ was reported [77]. It would be necessary to carry out some experiments in continuous operative conditions at a full-scale that best represents the effective application and energy consumption of US process in a real working WWTP. In this way, the cost-benefit analysis could be more reliable, and it could be considered whether the upgrade of the US technology would be useful.

Another advantage in the use of US coupled to UV can be the cleaning action performed by the US on UV quartz lamps, a factor that can prevent fouling and maintains the disinfectant action of UV radiation more efficient [80,89]. Vasilyak [51], on the other hand, presents a more critical thought, stating that the application of US for cleaning quartz sleeves of UV lamps cannot replace chemical or mechanical cleaning traditionally used at real scale.

Table 3 shows the main sonophotolysis disinfection experiments carried out in recent years on synthetic or authentic water. It is necessary to deepen some aspects such as the synergy effect of the US with UV, confirmed by Jin et al. [62] but denied by Vasilyak [51], and if there is an effective advantage in applying the process on a large scale in a simultaneous or sequential treatment with UV. There are not many studies in the scientific literature that deal with the use of this technology applied together with UV rays and only a few experiments concern the pilot scale up to about 80–100 L, however more representative of laboratory reactors. The application of US on real WW needs to be further investigated and its combined application with biological treatment merits further research to optimize the technical efficiency and the cost as well.

### 4.2. Photocatalysis

The photocatalysis (PC) process is based on the principle of photo-excitation of a semiconductor oxide upon absorption of light radiation [91]. Electrons (e^−^_CB_) in the valence band of the semiconductor are excited to the conduction band, leaving a positive hole (h^+^_VB_) in the valence band [92]. The electrons and holes formed during this process are involved in the redox reactions: oxidation of water molecule by the hole in the valence band brings to the production of highly active reactive oxygen species (ROS) [93,94] such as OH^•^ having a very high redox potential (OH/H_2_O 2.80 V), compared to other oxidizing agents, such as ozone (O_3_/O_2_, H_2_O 2.07 V) [95]. In the mechanism of disinfection, OH^•^ radicals attack the bacterial cell wall. The chances of radicals reaching intracellular components such as DNA are slim because they can only travel short distances. Their chances of damaging DNA are increased when they are generated near the target cells. The attack of intracellular components can only occur through the generation of other oxidants, such as lipid radicals, hydrogen peroxide and superoxide [96].

Many different materials can be used as semiconductors for PC such as titanium dioxide (TiO_2_), zinc oxide (ZnO), magnesium oxide (MgO), calcium oxide (CaO), tin oxide (SnO_2_), tungsten oxide (WO_3_), iron oxide (Fe_2_O_3_) and aluminum oxide (Al_2_O_3_) [94,97]. In recent years, these semiconductors have been extensively investigated coupled with UV for water disinfection [98,99,100,101,102]. Among these, TiO_2_ is widely used in photocatalytic processes, due to its advantages: ability to absorb solar radiation, chemical long-term stability, non-toxicity, and low cost [93,103]. TiO_2_ also has not negligible drawbacks which have limited its use in large industrial applications such as difficult separation of the powder from the treated water solution [27,104] and generation of visible light absorbance decreased [105].

Different strategies have been developed to enhance the photocatalytic efficiency with the modification of photocatalyst [97] such as mesoporous supports, metal doping, non-metal doping, nanoparticles, semiconductor coupling [106,107]. To overcome these operational problems caused by suspensions of fine powder, catalysts are usually immobilized on different supports such as silica gel [108], alumina [27], activated carbon [109], polymers [88], glasses [106], meshes [110,111] and graphene oxides [100].

Baogang Zhang et al. [97] examined the photocatalytic disinfection performance of various carbon supported Vanadium tetrasulfide (VS_4_) nanocomposites based on the bacteria inactivation rate of *E. coli* as an indicator. Among them, the cost-effective and lattice-structure VS_4_/CP (carbon powder) showed the best disinfection performance for removing *E. coli* under both simulated visible light (irradiance:100 W m^−2^, dose: 18 J cm^−2^) and sunlight (irradiance: ~379.2 W m^−2^, dose: ~71.5 J cm^−2^), with a maximum inactivation rate of 9.7 log at 0.1 g L^−1^ of catalyst dosage and 9.6 × 10^9^ CFU mL^−1^ of initial *E. coli* densities, in 30 min. The large-scale application of photocatalytic disinfection process is also prevented by the difficulty of obtaining photocatalysts and their purchase cost [112]. However, the growing use of support materials is partly solving the separation and reuse mechanisms of photocatalysts, making it possible to apply it on a WWTP [113]. Future research must focus on the study of economically sustainable, easily available, reusable, regenerable and low environmental impact semiconductor materials with a minimum waste of resources.

TiO_2_ has a band-gap in the range 3.0–3.2 eV, and the goal of the future research is to find applications that best allow extending the light absorption of the photocatalysts from the UV range (~5% of solar radiation) to the visible range (45% of solar radiation). The direct use of solar radiation reduces the use of electricity otherwise necessary for UV lamps: a greener technology is developed [39,106]. For example, nano-based photocatalyst, can use the large fraction of the solar spectrum to generate powerful ROS [114,115]. Sreeja and Shetty K [116], in their tests with a laboratory *E. coli* cell culture, obtained a complete disinfection of 40 × 10^8^ CFU mL^−1^ *E. coli* cells in 15 min exploiting solar PC with 0.4 g/L Ag core-TiO_2_ shell structured (Ag@TiO_2_) nanoparticles. In recent years, PC processes using solar light attracted high interest [106,117] resulting highly cost-effective and sustainable [114] in particular for large-scale applications [103]. Most of the studies with solar light is limited to the pilot stage of a solar compound parabolic collector reactor. The total volume of the photo-reactor was 10 L in the applications of Agulló-Barceló et al. [118] and Booshehri et al. [119] with an illuminated volume of 4.5 L and 4.7 L, respectively. Ferro et al. [120] used a pilot-scale photoreactor with a volume of 8.5 L and an illuminated volume of 4.7 L. In all cases the irradiated collector surface was 0.4 m^2^. The experiments reported in Table 3 were carried out in batch reactors with liter-scale treatment capacities. It is important to focus future studies on the exploitation of sunlight on a wider scale to understand the possibility of its effective potential in a treatment at a real WWTP.

Another application is the metal doping that introduces metal ions into a pure semiconductor to change its electronic properties and enhance photocatalytic efficiency [27]. The dopant shifts the absorption to the visible wavelengths by substituting titanium (referring to TiO_2_) in the substitutional or interstitial sites. Furthermore, dopant addition modifies particle size and crystal structure of the material [39]. However, the doping procedure is expensive and complicated, preventing its practical application and wide-ranging use [97]. Research should focus more on this critical aspect with the aim of identifying cheaper and therefore implementable technologies in an industrialized management reality.

Meng et al. [121] investigated another important advantage of the PC process in the treatment of biologically treated municipal WW in their study. UV/TiO_2_ PC has been found to simplify the high molecular weight precursors of THMs into smaller molecules like volatile organic acids. PC, therefore, possibly placed before a chlorination treatment, reduces the formation of disinfection by-products, such as THM, a cause of changes in the characteristics of the dissolved NOM. Therefore, as reported in Table 4, synthetic matrices have also often been used, obtained from cell cultures of the target bacterium to be tested. The results acquired from real WW samples are certainly more reliable because it is a complex of many substances that can influence not only ROS but also the physicochemical properties of photocatalysts. This mechanism of influence is still unclear, but in order to encourage the use of photocatalytic processes in large-scale applications, it is necessary to experiment their performance with real matrices.

### 4.3. Photoelectrocatalysis

Photoelectrocatalysis (PEC) is an advanced oxidation process that involves the combination of conventional PC with the use of electricity [126]. The performance of the PC treatment can be adversely affected by the rapid recombination of the photogenerated electrons and holes (e^−^_CB_/h^+^_VB_) [47,127] because each recombination leads to the loss of a hole that otherwise would have been a precursor of a disinfection reaction. Several studies suggested that, applying a constant current density or a constant bias anodic potential to the illuminated semiconductor prevents this recombination [14,15]. As demonstrated by Mesones et al. [128], the production of oxidizing species is directly proportional to the current density applied to the anode. Without electron donors or acceptors, the electron-hole pairs can recombine to release heat or migrate to the surface of the semiconductor and react with species that have been adsorbed there [129].

Some works have reported more efficient degradation of bacteria by PEC compared to UV or PC [47,130,131] under similar experimental conditions. The lab-scale experiments of Nie et al. [132] showed that For *E. coli* K-12 all bacterial cells were completely killed by the PEC process within 180 s, whereas only ca. 0.5-log reduction of bacterial cells was achieved with PC treatment, even with a time of 370 s. PEC inactivation was more effective thanks to the greater utilization of photo-generated holes.

As in the case of PC, it would be interesting to test materials that can be activated with a wavelength equal to that emitted by the solar spectrum, thus saving on the use of UV lamps. Absorbing light in the visible region of the solar spectrum is more sustainable both from an economic and an environmental point of view [133]. In fact, some researchers [134,135,136] focused their attention on visible light by simulating solar radiation with xenon lamps, as a possible option to induce the desired reactions to the semiconductor. However, further research studies are needed to identify new technologies that allow overcoming some operative problems related to the absorption of visible light, such as the excessive timing required for inactivation during cloudy days, the low flow of treated water, the presence of spores and viruses resistant to treatment and disinfection efficiency adversely affected by the turbidity of the water [18]. The doping of semiconductors with cations or anions is carried out with the aim of obtaining photoactive materials with greater reactivity in the visible part of the solar spectrum, given that UV corresponds to about 5% of sunlight [137].

In Table 5 several results of PEC applications aimed to disinfect both real and synthetic waters are reported. The tested matrices are mostly synthetic waters presenting a model bacterium inside to evaluate the inactivation efficiency of the considered photoanode. On the contrary, Venieri et al. [136] performed a series of disinfection tests with real effluent collected from the effluent of the activated sludge treatment of a municipal WWTP. In these tests, unlike those with samples containing only selected bacterial populations, a higher value of applied electric potential was chosen (5 V instead of 3 V). The raising of the anodic potential was made to improve the inactivation rate of the process since the aqueous matrix consists of a real discharge more complex and heterogeneous sample as it has presented several organic and inorganic compounds that can interfere with the disinfection process. Part of the ROS generated with PEC can react with the organic carbon and with the bicarbonates, sulfates and chlorides present in WW. In general, in a real effluent will occur a “consumption” of ROS which are not used for the inactivation of pathogens. Therefore, further studies on real waters would be needed to deepen this aspect, to quantify in more detail the optimal anodic potential that must be applied.

Despite several tests were conducted, the experimental scale was always at laboratory scale, no full-scale applications are reported in literature. In all the experiments, small reactors with a volume between 0.05 and 1 L were used and the results obtained on this scale may be less reliable and representative compared to applications involving the use of greater quantities of water. Several aspects that could happen at the full-scale such as mass transfer phenomena are overlooked. Therefore, the industrialization of this process has not yet taken place and is prevented by various problems such as the important investment and operative costs and the management complexity that requires qualified personnel [138]. Moreover, different models of reactors must be studied and developed to obtain an acceptable process efficiency and to make the PEC technologies applicable to a treatment scale level of industrial WWTP [137]. Wu et al. [139] developed a novel integrated system comprising three-dimensional electrochemical reactors and three-dimensional biofilm electrode reactors in series for coking WW treatment to improve removal efficiency and to save energy consumption. Results indicated an energy consumption of 15.6 kWh m^−3^. Mesones et al. [128] evaluated the PEC inactivation of *E. coli* in water using a novel three-dimensional electrochemical reactor designed with a commercial anode of RuO_x_/Ti and an illuminated photocatalyst of GAC-TiO_2_ composite as a bipolar electrode. In the case of GAC incorporation, there was an improvement in the energy efficiency (energy consumption of 0.004 kWh m^−3^) in comparison to the pure electrolytic process with the same current density alone with an energy consumption of 0.014 kWh m^−3^. In the case of PEC processes, although the energy efficiency increased as the applied current density increased, a higher energy consumption has been obtained (6.89, 4.23 and 2.24 kWh m^−3^ for 0.03, 0.06 and 0.10 mA cm^−2^, respectively) compared to the electrochemical disinfection process only, due to the high electrical consumption of the UV-A lamp.

## 5. Discussion and Future Outlooks

According to the definition promoted by the EPA (2019) [140], the purpose of green engineering is to invest in sustainable technologies that reduce pollution and waste of resources, always guaranteeing the protection of human health and the environment. The high concentration of pathogen microorganisms is one of the main aspects that prevent the reuse of the water leaving the WWTPs. A unique solution for WW disinfection does not exist; the appropriate disinfection technology should be chosen case-by-case for each WWTP, simultaneously considering performance, economic profit, sustainability criteria, and destination of the effluent. The aspects to be evaluated to identify a sustainable disinfection technology for WW are (i) constructive and operational simplicity, (ii) efficiency in reducing pathogenic microorganisms, (iii) capital and management cost, (iv) minimization of by-products, (v) additional treatments, (vi) environmental impact, (vii) safety risk [12]. It is important and urgent to find and use alternative methods to traditional chlorine disinfection, the toxicity of which has been widely underlined and demonstrated replacing chemical treatments with the application of multi-barrier processes that allow achieving a low environmental impact [31]. Following the prospects of reuse water in a circular economy perspective, the ecological nature of the UV technique (no DBPs were formed) and its simultaneous applications with US, different photocatalysts, and electric current makes it a promising candidate for the WW disinfection process. The presented review begins by critically analyzing the UV disinfection applied individually to the WW treatment and continues by presenting the UV treatment in combination with different non-chemical technologies.

As can be seen from the analysis of publications, in recent years, scientific literature has been focusing mainly on UV-combined processes due to a series of strengths compared to UV alone.

Sonophotolysis, thanks to the additional action of ultrasound, especially upstream of UV, can solve the drawback of turbidity by acting mainly on the size of the suspended particles that made disinfection inefficient. However, the high energy consumption due to the US has inhibited the intensive use of this technology on a larger scale. This aspect is reflected in the few experiments published in the scientific literature conducted by researchers mainly at the laboratory scale or at most with pilot plants. Research on studies in continuous operative conditions at a larger scale should be further studied to better prefigure the application and actual energy consumption of the sonophotolysis process in a daily functioning system. The aim would be to have a more reliable cost-benefit analysis to better understand the possible advantage of implementing the technology in a WWTP.

PC thanks to the production of ROS attack the intracellular material, leading to a greater log-reduction of microorganisms compared to previous treatments. If the semiconductors used are in the form of powders there is a difficult to filter them by treated water, and in general, photocatalysts can show a low absorbance in the visible light field. To overcome these operational problems different strategies have been developed with the modification of photocatalyst such as mesoporous supports, metal and non-metal doping, nanoparticles, and semiconductor coupling. The possibility of exploiting solar visible light is also a promising aspect but one that still needs further researchers to allow its effective application. The difficulty of finding suitable photocatalysts, the purchase cost of photocatalysts, the cost and complexity of procedures such as doping, the rapid recombination of the photogenerated electrons and holes that can adversely affect the performance of the treatment, are all weakness which have limited the PC implementation in large industrial applications. Moreover, several ROS generated can react with different composts present in a real effluent with a “consumption” of radicals not used for the inactivation of pathogens. Further studies on real waters would be needed to deepen this aspect, to quantify in more detail the optimal anodic potential that must be applied.

PEC process presents a more efficient degradation of bacteria compared to UV or PC thanks to the greater utilization of photo-generated holes preventing this recombination from applying a current density to the photocatalyst. It allows in a short time to remove up to almost all microorganisms. On the other hand, PEC presents the same problems as the PC about the type and method of use of semiconductors. Despite several tests were conducted both for PC and PEC, synthetic matrices have often been used, obtained from cell cultures of the target bacterium tested and no full-scale applications are reported in literature. The mechanism of influence is still unclear, but the results acquired from real WW samples are certainly more reliable because authentic water is a complex of many substances that can influence not only ROS but also the physicochemical properties of photocatalysts. It is also necessary to deepen research on different reactor configurations with larger volumes to further increase the efficiency of both PC and PEC processes and to make these technologies applicable to a treatment scale level of industrial WWTP.

Referring to the combined processes with UV (US + UV, PC, PEC), to compare the different technologies and identify the most potentially interesting ones in terms of sustainability, specific energy consumption could be considered as a possible aspect of comparison. It is one of the most important aspects at full-scale and one of the most decisive when choosing the plant treatment chain. In this case, the limiting factor is the lack of studies of these technologies on full-scale. the energy consumptions supplied refer to laboratory-scale experiments and evaluate possible costs in case of a real scale application is very difficult. Moreover, in the scientific literature the information on this aspect is limited. Only for UV applied individually was a specific energy consumption relative to the full--scale (0.04–0.13 kWh m^−3^) [59], while for the other treatments data on the pilot scale were found: 5.35 kWh m^−3^ for sonophotolysis [85] and 2.24–6.89 kWh m^−3^ for photoelectrocatalysis [128].

In general, future studies should focus mainly on aspects such as (i) use of real WWTPs effluents, (ii) the research of easily available, cheaper, reusable, regenerable different photocatalyst materials without the release of dangerous substances into the water, (iii) the study of catalysts that are activated thanks to wavelengths emitted by the solar spectrum, (iv) optimization of the design of reactors, at least at the pilot-scale, to increase knowledge on the process yields, contact times and energy costs. Only by deepening these technologies on different aspects and acquiring a greater awareness of their operative functioning, it will be possible to transfer their application to the industrial scale of a WWTPs.

In conclusion, as also shown by Collivignarelli et al. [141], the recent CoViD-19 has brought to light a structural lack of studies on the inactivation capacity of viruses (especially coronaviruses) in real aqueous matrices by means of disinfectants such as UV. Also, in this area further research is strongly suggested.

## 6. Conclusions

Among the critical issues that prevent the reuse of WWTPs effluents, the microbiological component plays a key role. A unique solution for WW disinfection does not exist but the appropriate disinfection technology should be chosen case-by-case for each WWTP, taken into consideration also the impact on the environment. More than 130 publications on UV-based treatments (without the formation of DBPs) are reviewed discussing their strengths and critical aspects. UV-based combined processes in WWTPs for reuse of their purified effluents present excellent prospects thanks to an absent environmental impact in terms of DBPs formation and very high disinfection yields (in most cases higher than 3-log reduction). To date the main limits to the limited application on a larger scale (or absent in the case of UV-based combined processes) are: (i) the high capital and operative costs, (ii) the limited or absent experience of full-scale plant management (especially for UV-based combined processes), and (iii) the limited literature on certain processes (e.g., PEC) mainly based on tests on synthetic waters in laboratory tests despite real waters with very low scale reactor. Therefore, further in-depth studies are required to ensure full applicability of UV-based combined processes in WWTPs for reuse of their purified effluents.

## Figures and Tables

**Table 1:** Several results of UV disinfection tests. S: synthetic water, R: real water, WW: wastewater, WWTP: wastewater treatment plant.

Type of Water	Characteristic of Water Tested	Operative Condition	Experimental Scale	Results			References
R	Primary (I) and secondary (II) WW effluents	35 J m^−2^ (I) 62 J m^−2^ (II)	Lab scale reactor (0.05 L)	*E. coli*: 1-log			[44]
S	Bacteria suspension in phosphate buffered saline solution	8 mJ cm^−2^	Bench scale reactor (0.01 L)	*E. coli*: >4.5-log			[60]
S	Bacteria suspension in sterilized saline solution (10^5^ CFU mL^−1^)	15 mJ cm^−2^	Lab scale reactor (0.015 L)	*E. coli*: >4.5-log ^a^			[56]
R	Secondary effluent of municipal WWTP	10 mJ cm^−2^	Lab scale reactor (0.015 L)	*Total coliform*: >2-log ^a^			[56]
R	Primary (I) and secondary (II) effluents of pilot WWTP	50 mJ cm^−2^	Lab scale reactor (0.02 L)		*P. aeruginosa* (Strain 1)	*P. aeruginosa* (Strain 2)	[61]
				I	1-log	2-log	
				II	2-log	3-log	
R	Secondary effluent of municipal WWTP	30 mJ cm^−2^	Bench scale reactor (3 L)	*E. coli*: 3.4-log			[62]
S	Bacteria suspension in sterilized saline solution (10^7^ CFU mL^−1^)	40 mJ cm^−2^	Lab scale reactor (0.015 L)	*E. coli*^b^: >5.5-log			[63]
S	Bacteria suspension in phosphate buffered saline solution (10^7^ CFU mL^−1^)	80 mJ cm^−2^	Lab scale reactor (0.04 L)	*E. coli*^c^: 6-log			[48]

^a^ There was no difference in inactivation between low- and medium-pressure UV lamps. ^b^ *E. coli* CGMCC 1.1595. ^c^ Antibiotic-resistant SER2 strain isolated from secondary effluents of WW treatment.

**Table 2:** Several results of PUV and UV-LED disinfection tests. S: synthetic water, R: real water.

Type of Water	Type of Technology	Characteristic of Water Tested	Operative Condition		Experimental Scale	Results		References
S	PUV	Bacteria suspension in phosphate buffered saline solution	3 mJ cm^−2^		Bench scale reactor (0.01 L)	*E. coli*: 4.26-log Phage T4: 4.29-log Phage T7: 2.72-log		[60]
S	PUV	Distilled water with organic dairy WW solids (25 mg L^−1^) and 1 × 10^6^ CFU mL^−1^ initial concentration of *E Coli*	1946 mJ cm^−2^		Bench scale reactor (5 L)	*E. coli*: >3.5-log		[64]
S	PUV	Bacteria suspension in phosphate buffered saline solution	PL1: 76 J cm^−2 a^ PL2: 95 J cm^−2 a^		Lab scale reactor (0.003 L)	*E. coli*: 11-log (PL1) *E. coli*: 6-log (PL2)		[66]
S	UV-LED	Bacteria suspension in sterile saline solution (10^7^ CFU mL^−1^)	265 nm LED: 10.91 ± 0.76 mJ cm^−2^ (265 + 280) nm (50%): 12.57 ± 0.81 mJ cm^−2^ (265 + 280) nm (75%): 13.78 ± 0.67 mJ cm^−2^ 280 nm LED: 15.35 ± 1.52 mJ cm^−2^		Lab scale reactor (0.005 L)	*E. coli*: 4.5-log		[67]
S, R	UV-LED	Deionized water, kaoline suspension (DIK), secondary effluents of urban WWTP (SE)	30 mJ cm^−2^ Exposure time: 900 s		Batch reactor (0.03 L)	*E. coli*: >4.5-log (DIK), 3-log (SE)		[68]
R	UV-LED	Domestic WW from a sewer system, the treated with settler and sand filter	69.4 mJ cm^−2^ Exposure time: 412 s Flow rate: 10 mL min^−1^		Flow through reactor (0.0686 L)	MS2 coliphage ^b^: 3.7 ± 0.2 -log		[69]
R	UV-LED	Secondary effluent of WWTP with UASB treatment ^c^	Wavelength (nm)	Irradiance intensity (mW cm^−2^)	Lab scale reactor (0.02 L)	Wavelength (nm)	*E. coli* inactivation log	[70]
			255	0.017		255	2.6	
			280	0.019		280	4.0	
			365	0.004		255/280	3.7	
			405	0.077		255/280/405	3.8	
			Exposure time: 15 min			280/365/405	3.5	
						255/280/365/405	3.8	

^a^ Two spiral lamps were used, PL1 and PL2, with wavelength cut-offs of 190 and 240 nm, respectively. ^b^ In this study, pure cultured male specific 2 (MS2) coliphage was used as the representative microorganism and biodosimeter. ^c^ The treated wastewater used in this research was collected from a WWTP (flow average rate 80 Ls^−1^, BOD average removal efficiency 90%) which consists of an upflow anaerobic sludge blanket (UASB) followed by trickling filter and circular clarifiers.

**Table 3:** Several results of sonophotolysis disinfection tests. S: synthetic water, R: real water, UV: ultraviolet radiation, US: ultrasound, t: contact time.

Type of Water	Characteristic of Water Tested	Operative Condition	Experimental Scale	Results	References
R	Secondary effluents of municipal WWTP	US: 310 W L^−1^ UV: 0.037 mJ cm^−2^ t (US): 10 s t (UV): 30 s	Lab scale reactor	*E. coli*: >5.5 log *Feacal streptococci*: >6.5 log	[86]
R	Tertiary effluents of domestic and industrial WWTP ^a^	US: 350 W UV: 1656 mJ cm^−2^ t:15 min	Pilot plant (80 L)	*E. coli*: 1.6-log Total coliform: 1.7-log	[80]
		US: 1400 W UV: 1656 mJ cm^−2^ t:15 min		*E. coli*: >4-log Total coliform: 3.9-log	
R	Secondary effluents of municipal WWTP	US: 180 W, 40 kHz, 2.64 kJ L^−1^ UV: 30 mJ cm^−2^	Bench-scale reactor (3 L)	*E. coli*: 5.4-log	[62]
R	Secondary effluents of municipal WWTP	US: 89.9 W UV: 174 W	Pilot plant (1200 L h^−1^, 96 L)	Faecal coliform: 4.24-log	[85]
R	Secondary effluents of municipal WWTP	US: 100 W UV: 170 W	Pilot plant (96 L)	Total coliform: 3.87-log	[77]
S, R	Deionized water, kaoline suspension (DIK), secondary effluents of urban WWTP (SE)	US: 33 kHz, 200 W UV-LED: 30 mJ cm^−2^ t (US): 40 s t (UV): 900 s	Batch reactor (US: 2 L, UV: 30 mL)	*E. coli*: 6-log (DIK), 3.5-log (SE) ^b^	[68]
R	Secondary effluents of municipal WWTP	US: 130 kHz UV: 600 mJ cm^−2^ t: 240 min	Lab scale reactor (US: 50 mL, UV:20 mL)	Sulfonamide resistant *E. coli*: 3.8-log Tetracycline resistant *E. coli*: 4.4-log	[90]

^a^ A pilot plant was installed downstream of the full-scale sand filtration unit of the WWTP. ^b^ The results reported were obtained from the graphic representation.

**Table 4:** Several results of PC disinfection tests. S: synthetic water, R: real water, UV: ultraviolet radiation, t: contact time.

Type of Water	Characteristics of Water Tested	Photocatalyst	Operative Condition	Experimental Scale	Results					References
S	Bacteria suspension in sterilized saline solution (10^7^ CFU mL^−1^)	g-C_3_N_4_/TiO_2_	Xe lamp with UV filter: 30 mW cm^−2^ t: 180 min	Lab scale reactor (0.11 L)	*E. coli*: 100% inactivation ^a^					[17]
S	Bacteria suspension in sterilized saline solution (2.42 × 10^6^ CFU mL^−1^)	TiO_2_	UV: 42.7 mJ cm^−2^	Pilot plant (1000 L h^−1^)	*E. coli*: 3.05-log					[40]
R	Secondary effluents of municipal WWTP (1.5 × 10^8^ CFU mL^−1^)	0.1% Mn:TiO_2_ 0.1% Co:TiO_2_ 0.04 Mn/Co:TiO_2_	Xe O_3_-free lamp: 1.31 10^−2^ W m^−2^ t: 90 min	Lab scale reactor (0.3 L)	*K. pneumoniae*					[122]
					Sunlight		UV			
			Sunlight: 12.7–13.4 W m^−2^ T: 29–32.7 °C t: 60 min		0.1% Mn:TiO_2_	1-log	4-log			
					0.1% Co:TiO_2_	2-log	6-log			
					0.04 Mn/Co:TiO_2_	1-log	6-log			
R	Secondary effluents of municipal WWTP (*E. coli*: 200 CFU mL^−1^, *Salmonella ssp.*: 159 CFU ml^−1^, *Shigella ssp*.: 95 CFU ml^−1^, *Vibrio cholerae*: 10 CFU ml^−1^)	undoped TiO_2_ Ag-doped TiO_2_ Cu.doped TiO_2_ Fe-doped TiO_2_	UV: 70 mW cm^−2^ t:15 min	Lab scale reactor (0.7 L)		*E. coli*	Salmonella species	Shigella species	Vibrio cholerae	[39]
					undoped TiO_2_	0.757-log	0.724-log	0.802-log	0.51-log	
					Ag-doped TiO_2_	1-log	1.025-log	1.003-log	0.6-log	
					Cu-doped TiO_2_	0.903-log	0.9-log	0.978-log	0.45-log	
					Fe-doped TiO_2_	1-log	0.754-log	0.45-log	0.55-log	
S	Bacteria suspension in sterilized saline solution (5 × 10^6^ CFU mL^−1^)	Ag@ZnO core-shell nanoparticles	Sunlight: 90.000 ± 5000 lux T: 35°C t (*E. coli*): 60 min t (*S. aureus*): 90 min	Lab scale reactor (2 L)	*E. coli*: 6-log *S. aureus*: 6-log					[123]
R	Secondary effluents of municipal WWTP (300 ± 30 CFU mL^−1^)	N-TiO_2_/PS ^b^	LED: 81.6 W t: 120 min	Lab scale reactor (0.5 L)	*E. coli*: 1.13-log ^c^					[106]
R	Secondary effluents of WWTP (10^7^ CFU mL^−1^)	CoFe_2_O_4_/HTCC ^d^	Xe lamp: 300 W t: 160 min	Lab scale reactor (0.020 L)	*E. coli*: 7-log					[112]
R	Secondary effluents of local WWTP (4.5 × 10^7^ CFU mL^−1^)	AgFeNTFS ^e^	Fluorescent lamp: 330 W m^−2^ t: 90 min	Lab scale reactor	*E. coli*: 3-log					[124]
S	Bacteria suspension in phosphate buffered saline solution (10^6^ CFU mL^−1^)	CNCT-3 ^f^	Xe lamp: 50 mW cm^−2^ t: 90 min	Lab scale reactor (0.01 L)	*E. coli*: 6-log					[125]

^a^ In the text is expressed as complete inactivation. ^b^ The PS spheres were used as substrate for the deposition of N-TiO_2_ powder. ^c^ The value is expressed as a percentage as a result in the corresponding research study. ^d^ Hydrothermal carbonation carbon (HTCC)-coated cobalt ferrite (CoFe_2_O_4_) composites with HTCC coating thicknesses between 0.62 and 4.38 nm. ^e^ Magnetic photocatalyst Ag/Fe, N-TiO_2_/Fe_3_O4@SiO_2_ was synthesized through a multi-step method by codoping Fe and N in the TiO_2_-based component, Ag deposition and equipping the Fe_3_O_4_@SiO_2_ magnetic core. ^f^ Heterostructured g-C_3_N_4_@Co-TiO_2_ (CNCT) nanofibrous membranes fabricated by an electrospinning approach and subsequent thermal polymerization process. In the CNCT-3 sample the loaded melamine powder was 0.3 g.

**Table 5:** Several result of PEC disinfection tests. S: synthetic water, R: real water, UV: ultraviolet radiation, t: contact time.

Type of Water	Characteristic of Water Tested	Operative Condition	Experimental Scale	Results	References
S	Cell density of 10^7^ CFU mL^−1^ with Na_2_SO_4_ solution	TiO_2_/Ti-film Zirconium cathode Xenon lamp: 150 W, 1.31 × 10^−2^ W m^−2^ Electric potential: +2.0 V t: 120 min	Lab scale reactor (0.06 L)	*E. coli*: 7-log	[136]
R	Secondary effluent of municipal WWTP with cell density of 10^7^ CFU mL^−1^ and Na_2_SO_4_	TiO_2_/Ti-film Zirconium cathode Xenon lamp: 150 W, 1.31 × 10^−2^ W m^−2^ Electric potential: +5.0 V t: 90 min		*E. coli*: 100% inactivation ^d^	
S	Cell density of 7 × 10^4^ CFU mL^−1^ with Na_2_SO_4_ solution	Ti/TiO_2_–Ag nanotubes Pt gauze UV: 125 W, 9.23 W m^−2^ Xenon lamp: 150 W Electric potential: +1.5 V Ag/AgCl (3 M KCl) t (UV): 3 min t (visible irradiation): 30 min	Lab scale reactor (0.25 L)	*M. smegmati* UV: total inactivation Visible irradiation: 2.4-log ^c^	[134]
S	Cell density of 10^7^ CFU mL^−1^ with NaNO_3_ solution	TiO_2_ nanotubes Platinum foil UV: 28 mW cm^−2^ Electric potential. +1.0 V Ag/AgCl t (*E. coli* K12): 3 min t (*E. coli* BW25113 ^a^): 6.2 min	Lab scale reactor	*E. coli* K-12: 100% inactivation ^d^ *E. coli* BW25113: 100% inactivation ^d^	[132]
S	Cell density of 10^7^ CFU mL^−1^ with NaNO_3_ solution	N-doped carbonaceous/TiO_2_ Platinum foil Electric potential: +1.0 V Ag/AgCl Xenon lamp: 15 mW cm^−2^ t: 30 min ^b^	Lab scale reactor (0.05 L)	*E. coli*: 100% inactivation ^d^	[135]
S	Cell density of 3 × 10^2^ CFU mL^−1^ with Na_2_SO_4_ solution	Ag-doped Ti/TiO_2_ UVA: 8 W, 0.49 mW cm^−2^ Electric potential: +1.5 V Ag/AgCl (1 mol L^−1^ KCl) t (*E. coli*): 10 min t (*S. aureus*): 60 min	Lab scale reactor (0.254 L)	*E. coli*: 100% inactivation ^d^ *S. aureus*: 100% inactivation ^d^	[67]
	Cell density of 3 × 10^2^ CFU mL^−1^ with Na_2_SO_4_ solution	non-doped Ti/TiO_2_ UVA: 8 W, 0.49 mW cm^−2^ Electric potential: +1.5 V Ag/AgCl (1 mol L^−1^ KCl) t: 60 min		*E. coli*: 0.33-log ^c^ *S. aureus*:0.25-log ^c^	
S	Cell density of 10^3^ CFU mL^−1^ with Na_2_SO_4_ solution	2 coating-TiO_2_/ITO Ni mesh UVA: 6 W, 0.47 W m^−2^ Electric potential: +1.4 V t: 140 min	Lab scale reactor (1 L)	*E. coli*: 100% inactivation ^d^	[131]
R	Secondary effluent of urban WWTP with cell density of 1.6 × 10^9^ CFU mL^−1^	Ag(4%)-TiO_2_ polished Al foil UVA: 4 W Electric Potential: +1.5 V t: 16 min	Lab scale reactor (0.05 L)	*Faecal coliform*: 100% inactivation ^d^	[130]
S	Cell density of 10^6^ CFU mL^−1^ with Na_2_SO_4_ solution	Ag(4%)-TiO_2_ UVA: 125 W Electric potential: +1.7 V Ag/AgCl (3 M KCl) t: 5 min (*P. aeruginosa*) t: 15 min (*B. atrophaeus*)	Lab scale reactor (0.1 L)	*P. aeruginosa*: 100% inactivation ^d^ *B. atrophaeus*: 100% inactivation ^d^	[15]

^a^*E. coli* BW25113 is the mutant of the ancestral *E. coli* K12. ^b^ This time of complete inactivation was reached with the composite photoanodes obtained from 120 °C hydrothermal-calcination treatment. A time of 40 and 50 min with the fabrication at 150 and 180 °C, respectively. ^c^ The value is expressed as a percentage as a result in the corresponding research study. ^d^ In the text is expressed as complete inactivation.

## Data Availability

No new data were created or analyzed in this study. Data sharing is not applicable to this article.

## Abbreviations

ARG	Antibiotic resistance gene
DBP	Disinfection by-product
eARG	extracellular antibiotic resistance gene
PC	Photocatalysis
PEC	Photoelectrocatalysis
PUV	Pulsed ultraviolet radiation
ROS	Reactive oxygen species
THM	Trihalomethane
US	Ultrasound
UV	Ultraviolet radiation
WW	Wastewater
WWTP	Wastewater treatment plant
